# Spin-Based Chiral Separations and the Importance of Molecule–Solvent Interactions

**DOI:** 10.1021/acs.jpcc.3c01159

**Published:** 2023-07-13

**Authors:** Yiyang Lu, Tian Qiu, Brian P. Bloom, Joseph E. Subotnik, David H. Waldeck

**Affiliations:** † Chemistry Department, University of Pittsburgh, Pittsburgh, Pennsylvania 15260, United States; ‡ Departments of Chemistry, University of Pennsylvania, Philadelphia, Pennsylvania 19104, United States

## Abstract

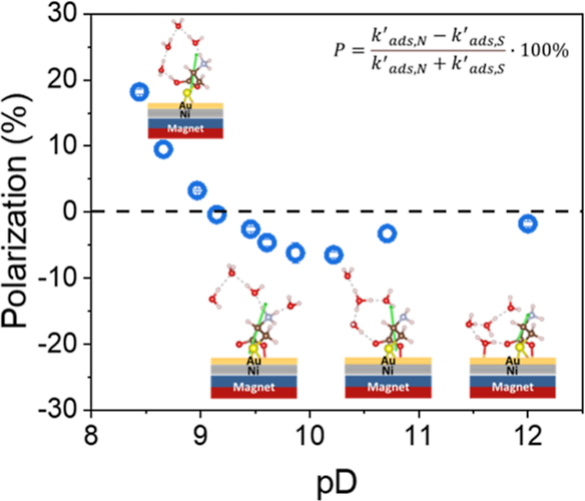

This work uses magneto-electrochemical quartz crystal microbalance methods to study the enantiospecific adsorption of chiral molecules onto a ferromagnetic substrate. The effects of solution conditions, pH, and solvent isotope composition indicate that the kinetics of the enantiomeric adsorption depend strongly on the charge state and geometry of the adsorbate, whereas no thermodynamic contributions to enantiospecificity are found. Density functional theory calculations reveal that an interplay between the adsorbate and solvent molecules is important for defining the observed enantiospecific preference with an applied magnetic field; however, it remains unclear if intermolecular vibrational couplings contribute to the phenomenon.

## Introduction

The chiral-induced spin selectivity (CISS) effect refers to the fact that chiral molecules and chiral materials (even if they are closed shell) display spin-dependent electronic responses.^1,2^ Early work on CISS emphasized the preference for electron propagation of a given spin through a chiral molecule with a given handedness;^3−5^ however, the discovery that charge-polarizing chiral molecules gives rise to a spin polarization in the molecule^6^ expands the implications and possible applications of CISS beyond that of simple spin filtering. For instance, Banerjee-Ghosh et al. showed that chiral molecules exhibit enantiospecific interactions with a magnetized ferromagnetic substrate and this process can be used for enantioselective separation from racemic solutions.^7^ More recently, Safari et al.^8^ showed that enantiospecific adsorption can occur at the single molecule level in ultra-high vacuum conditions. In related work, we, and others, have leveraged the enantiospecific interactions of chiral molecules with ferromagnetic substrates to perform electrochemically driven enantioselective redox reactions,^9−11^ to separate racemic solutions through substrate-driven crystallization,^12,13^ and to affect the helicity of polymers during electropolymerization.^14,15^

In our previous work,^16^ magnetic electrochemical quartz crystal microbalance (EQCM) studies revealed that the enantiospecific adsorption of l- and d-cysteine on a magnetized ferromagnetic substrate is a kinetically controlled process rather than a thermodynamic one, under ambient conditions. In addition, the enantiospecificity was found to depend strongly on the solution pH conditions. Because the binding geometry and ionization state of the molecule changes with pH,^17^ the variations in selectivity were hypothesized to arise from the geometry of the adsorbate. Indeed, Clever et al.^18^ have recently demonstrated the importance of molecular dipole moments in controlling the sign of the polarization in CISS-based measurements on oligopeptides, underscoring the importance of a molecule’s dipole moment direction relative to the surface. In other work, Tassinari et al.^13^ showed that d-asparagine (l-asparagine) preferentially crystallized on a ferromagnetic substrate with application of a north (south) magnetic field while the enantiopreference was reversed for glutamic acid; l-glutamic acid preferentially crystallizes on a ferromagnetic substrate with a north magnetic field and d-glutamic acid with a south magnetic field. These studies indicate that the enantiopreference is sensitive to the interaction conditions between the molecule and substrate and imply that they can vary on a molecule-to-molecule basis. That being said, not all studies have shown enantiospecific adsorption on ferromagnetic surfaces; Radetic and Gellman^19^ did not observe an enantiopreference in the adsorption of d- and l-aspartic acid on magnetized Ni(100) surfaces under ultra-high vacuum conditions. To fully exploit spin-mediated enantiospecificity for guiding chemical reactions, separations, and other processes, a more rigorous understanding of the physical properties which dictate enantioselectivity must be made. In particular, one essential question that remains unresolved is the effect of nuclear vibrations on spin selectivity as encapsulated through non-Born–Oppenheimer Berry forces; calculations on model Hamiltonians^20^ have suggested that such effects can be substantial in promoting CISS even with small spin–orbit couplings, but such calculations have not yet been run on realistic systems or Hamiltonians.

In this work, the enantiospecific adsorption of cysteine and *N*-acetyl cysteine methyl ester on a magnetized ferromagnetic substrate in H_2_O and D_2_O solutions was investigated and found to correlate with the geometry of the adsorbate on the substrate, the adsorbate’s ionization state (as determined by its p*K*_a_ value), and the solution conditions. Density functional theory (DFT) calculations corroborate these findings by revealing the importance of solvent interactions for achieving large differences in the adsorption kinetics of a pair of enantiomers.

## Methods

### Adsorption Kinetic Study with the EQCM System

The EQCM experiments were performed using a 7.9995 MHz quartz crystal with an EQCM cell attachment and a 430A potentiostat (CH Instruments). The surface area of the crystal is 0.205 cm^2^ and is coated with 100 nm nickel and 10 nm polycrystalline gold as the working electrode (CH Instruments). The counter electrode was Pt wire and the reference electrode was a saturated Ag/AgCl electrode.

The quartz crystal was first incubated in the cysteine solution for 1 h to allow the system to equilibrate before applying a potential. Cyclic voltammograms and the frequency response of the quartz were recorded for 30 cycles of the crystal to ensure that the surface was stable and the desorption and adsorption processes were reversible. Chronoamperometry experiments were performed immediately after the cyclic voltammetry acquisition with an initial potential of −0.8 V corresponding to reductive desorption of cysteine and then a more positive potential, −0.5 V, corresponding to oxidative adsorption. A 5 s pulse width was applied during the experiment and >150 cycles were performed to build up the statistics for the histogram plots; see Figure 2.

### Adsorption Isotherm Study with the EQCM System

Cyclic voltammetry and QCM measurements were used to build the data for the adsorption isotherms. The data were collected by scanning from −0.4 to −1.1 V versus saturated Ag|AgCl at a scan rate = 25 mV/s. The frequency change from the 30th cycle, corresponding to the oxidative adsorption of cysteine on a gold substrate, was converted to a change in mass (Δ*m*) according to the Sauerbrey equation by a factor of −1.4 ng/Hz. An excessive amount of cycles was necessary to allow the instrument to reach equilibrium and give consistent results. Next, the mass change (Δ*m*) was plotted versus concentration and fit to a Langmuir–Freundlich isotherm model.

### Optical Measurement

The optical data were collected in a 1 cm quartz cuvette. Absorbance spectroscopy was performed using an Agilent model 8453 spectrometer. Circular dichroism (CD) spectra were measured by using a JASCO J-810 CD spectrometer with a scan rate of 50 nm/min and a bandwidth of 1 nm. Each sample was scanned three times and the average of the three curves reported. Phosphate buffer with different pH values were used as blank solutions for baseline subtraction for different pH conditions.

### Calculation Methods

DFT calculations were performed using QUANTUM ESPRESSO (version 6.7.0).^21^ The generalized gradient approximation (GGA) of Perdew, Burke, and Ernzerhof (PBE)^22^ was used to treat electron exchange and correlation, with the D3 dispersion correction applied.^23^ The pseudopotentials for the elements Au, C, H, O, N, and S were chosen from the SSSP efficiency PBE pseudopotential database (version 1.1.2).^24^ For the system of interest, the plane-wave energy cutoff was tested and set to 65 Ry such that the pseudopotential error is less than 10 meV/atom. For cysteine adsorption and deprotonation, a slab model of the *p* (3 × 3) Au(111) surface with four Au layers and ∼18 Å of vacuum space was generated; for *N*-acetyl-l-cysteine methyl ester adsorption and deprotonation, a slab model of the *p* (4 × 4) Au(111) surface with three Au layers and ∼18 Å of vacuum space was generated. For all calculations, the bottom one layer of Au atoms was fixed and the rest of the atoms were allowed to relax. A dipole correction^25^ was added in the direction perpendicular to the Au surface. The Brillouin zone was sampled using a 4 × 4 × 1 shifted *k*-point grid. Explicit water molecules were added to each system near the hydrophilic functional groups, i.e., −SH, −NH_*x*_, and −COO(H). Besides the charge caused by deprotonation, a total of three electrons per cell were removed from the system for all cysteine-related calculations to mimic the electrode potential such that the cysteine molecule can reasonably adsorb on the Au surface. The dipole moment of the system (adsorbates + water molecules) is calculated using the nuclear charge and the Löwdin charge of all atoms excluding the Au atoms. In cases where the system is not neutral, the center of the net charge of the adsorbates + water molecules is chosen as the origin to calculate the dipole moment.

## Results and Discussion

**Figure 1:**
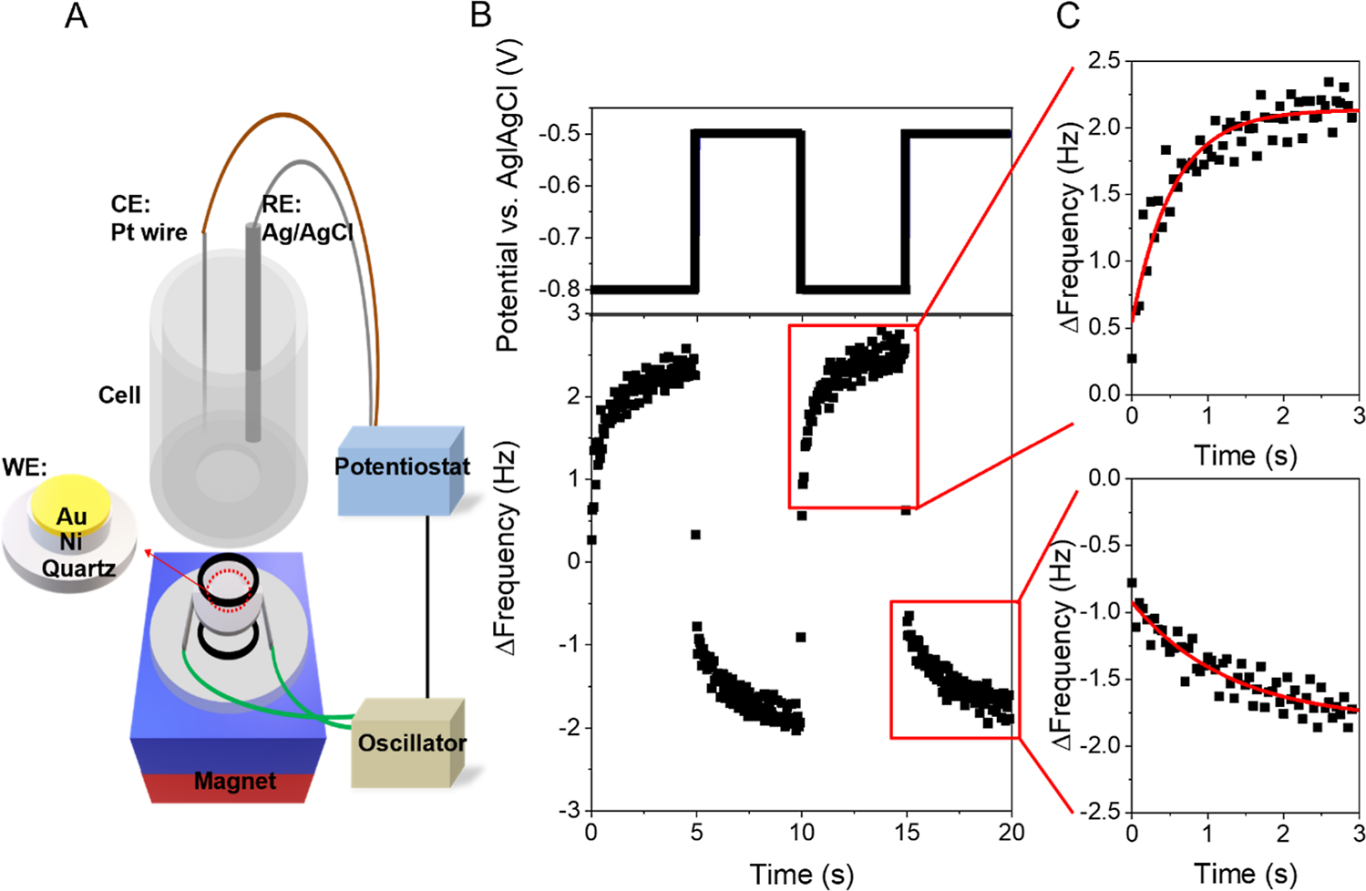
(A) Illustration of the EQCM setup in which a permanent magnet is placed underneath the quartz crystal electrode during measurements. The inset shows a zoomed-in rendering of the working electrode composition. (B) Representative chronoamperometry potential sequence (top) and corresponding frequency response of the quartz crystal electrode (bottom) used for an 8 μM solution of d-cysteine in a pH 8 buffer solution. (C) Expanded plot of the frequency response upon desorption (top) and adsorption (bottom) of d-cysteine. The red line is an exponential fit to the data used for extracting the effective rate constants.

**Figure 2:**
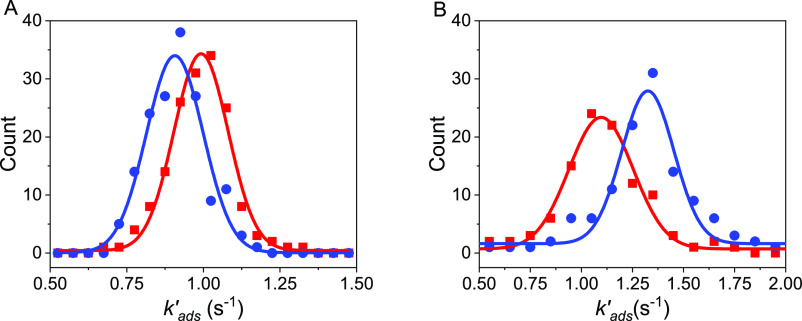
Histograms of the effective adsorption rate constant of an 8 μM solution of d-cysteine under a north magnetic field (red) and a south magnetic field (blue) for H_2_O (A) and D_2_O (B) at pH* = 8.5. A best fit to the data using a Gaussian distribution is shown by a solid line.

The effective adsorption rate of cysteine under different magnetization states (north versus south) was measured using time-dependent magnetic EQCM measurements, as described in our previous work;^16^ see Figure 1A for a scheme of the experimental method and the methods section for additional experimental details. Briefly, a quartz crystal was coated with a 100 nm Ni/10 nm Au film and used as the working electrode in a three-electrode electrochemical cell. The potential of the working electrode was varied, causing adsorption and desorption of the chiral molecule adlayer, and the time-dependent frequency response of the quartz crystal was recorded to quantify the mass change. A 0.54 T permanent magnet was placed underneath the quartz crystal for the entirety of the experiment to magnetize the electrode [see Figure 1A; north (south) denotes which magnetic pole is pointing toward the electrolyte along the normal to the Ni film’s surface]. The effect of the magnetization direction on the adsorption kinetics was then monitored. Figure 1B shows a graphical representation of the potential sequence (top), and the resulting change in frequency recorded by the quartz crystal (bottom) used for cysteine; the potential was cycled between −0.8 V to initiate cysteine desorption and −0.5 V to induce adsorption, in 5 s intervals. The frequency response within the first 3 s for each desorption (top) and adsorption (bottom) process is shown in Figure 1c , and the red line shows a best fit to the data with an exponential growth or decay. The time constant generated from the fit was then used to calculate an effective adsorption rate constant, *k*_ads_^′^, and a histogram of the rates from >150 adsorption events was compiled. The histograms were then fit by a Gaussian distribution to obtain an average *k*_ads_^′^ and standard deviation. Figure 2A shows representative histograms for 8 μM d-cysteine in a pH 8 buffer solution in H_2_O, under north (red) and south (blue) applied magnetic fields. The difference in peak positions of the Gaussian distributions shows the significant dependence of the adsorption of d-cysteine on the magnetization state of the electrode.

To experimentally explore the importance of vibrational contributions, a solution isotope dependence on the adsorption behavior of cysteine was investigated. Figure 2 shows histograms of *k*_ads_^′^ for 8 μM d-cysteine in H_2_O (A) and D_2_O (B) at pH* = 8.5, where pH* represents the reading from a pH meter. Thus, the pH in H_2_O is the same as pH*, but the pD in D_2_O is calculated by pH* + 0.45 in accordance with established protocols.^26^ Interestingly, for pH* = 8.5, *k*_ads_^′^ of d-cysteine is higher for a north magnetized electrode (red) than for a south magnetized electrode (blue) in H_2_O, however, the opposite is true in D_2_O; *k*_ads_^′^ is higher (lower) for a south (north) magnetized electrode.

To determine if the difference in adsorption behavior with solvent is rooted in a thermodynamically driven process, the Gibbs free energy for the adsorption of cysteine, Δ_ads_*G*, in D_2_O was measured; see Figure S3 and the corresponding discussion. The average Δ_ads_*G* of d-cysteine with a north magnetic field was −34.2 ± 1.4 kJ and for a south magnetic field was −34.6 ± 1.3 kJ, whereas the average Δ_ads_*G* for l-cysteine was −34.0 ± 1.1 and −34.2 ± 1.0 kJ with a north and south magnetic field, respectively. These results are consistent with the Δ_ads_*G* of d- and l-cysteine in H_2_O reported in our previous work^16^ and indicate that the solvent isotope does not significantly affect the thermodynamics of cysteine adsorption at pH* = 8.5. To further explore the effect of isotope and solution conditions on *k*_ads_^′^, a series of different pH/pD values were measured. The difference between the effective rate constant under north and south magnetization were quantified through a polarization parameter, *P*, defined as
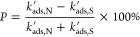
where *k*_ads,N_^′^ and *k*_ads,S_^′^ correspond to the adsorption rate constant under north and south applied magnetic fields, respectively.

**Figure 3:**
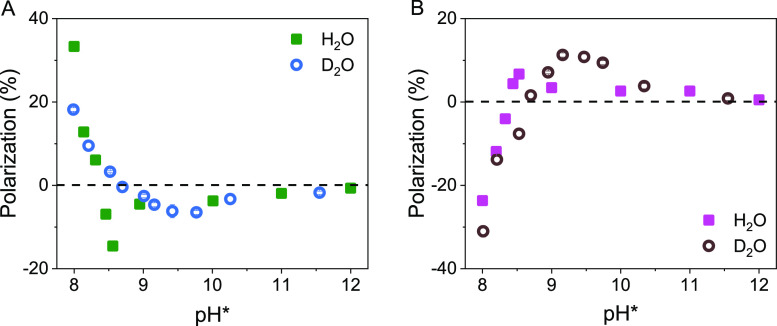
Polarizations in the adsorption rate constant in different pH* solutions for l-cysteine (A) in H_2_O (green square) and D_2_O (bluish graycircle) and d-cysteine (B) in H_2_O (purple square) and D_2_O (brown circle).

Figure 3 shows *P* for solutions of 8 μM l-cysteine (panel A) and d-cysteine (panel B) in H_2_O (squares) and D_2_O (circles) as a function of pH*. The l-cysteine and d-cysteine enantiomers display an opposite sign in the polarization at each pH* in both H_2_O and D_2_O solutions. The asymmetry in adsorption rates, as well as the inverse behavior of the enantiomers, is consistent with a CISS-mediated phenomenon.^1^ In H_2_O at pH = 8, the *P* is large and positive (33%) for l-cysteine, whereas the *P* is large and negative (−24%) for d-cysteine. As the pH is increased, the *P* for both l- and d-cysteine quickly transition to an opposite sign, reaching −15.6% for l-cysteine and 6.7% for d-cysteine at pH = 8.6, then decrease to zero at high pH. In D_2_O at pH* = 8, *P* for both l- and d-cysteine have a maximum value (18.5 and −31%, respectively), then gradually transition to a *P* with the opposite sign, reaching a maximum value at ∼pH* = 9.3 (−6.9% for l-cysteine and 13.8% for d-cysteine), then decrease to zero as the pH* is increased further. The crossover of the *P* for both d- and l-cysteine in H_2_O occurs at around pH* = 8.3, while in D_2_O it shifts to a higher pH* value, ∼8.6 (corresponding to pD = 9.05). The two crossover points correlate with the solvent-dependent p*K*_a_ value of the sulfur moiety of cysteine; ∼8.3 and ∼8.9 in H_2_O and D_2_O, respectively.^27−29^

**Scheme 1:**
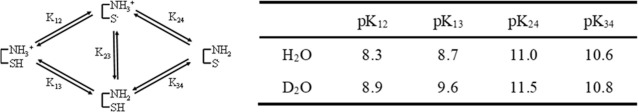
General Ionization Scheme (Left) and a Table of Ionization Constants (Right) for Cysteine in a Solution of H_2_O and D_2_O The data are taken from refs (27), (29), and (32).

Although the form of the polarization versus pH* curves are qualitatively the same in H_2_O and D_2_O, the *P* value undergoes a much sharper crossover at the thiol p*K*_a_ value in H_2_O than in D_2_O. One hypothesis is that this change in shape arises from the tautomerization equilibria involving sulfhydryl groups, which are subject to an inverse solvent deuterium isotope effect,^29^ whereby the zwitterion is preferentially stabilized in D_2_O relative to H_2_O. This is reflected experimentally in a larger p*K*_a_ window between pK_12_ and pK_13_ in D_2_O relative to H_2_O; see Scheme 1. When the pH is greater than 8.7 in H_2_O, or the pD is greater than 9.6 in D_2_O, the non-zwitterionic form of cysteine dominates. Compared to the zwitterionic form of cysteine, the non-zwitterionic form has a smaller dipole moment,^30^ which may result in a smaller CISS response, effectively decreasing the polarization.^31^ Thus, the differences in cysteine p*K*_a_ values could explain differences in polarization for H_2_O and D_2_O at pH* = 8.5 as shown in Figure 2; the dramatic crossover for H_2_O compared to the elongated crossover in D_2_O creates differences in the sign of the *P* at intermediate pH*. This hypothetical situation is almost certainly more complicated on the electrode surface, where due to the electrical double layer and the reduced mobility of the solvent molecules, the local electric field and geometries play important roles. In particular, our simulations suggest that the proton from the thiol group is activated when S binds to the Au surface, and a stable local solvation ring structure is formed around this proton. Thus, the four possible charge states depicted in Scheme 1 may be very different in the context of adsorbates (as described below).

Previous research^7,8^ indicates that the CISS properties of a molecule are often dictated by the enantiomer under investigation and correlate strongly with the sign and magnitude of the lowest energy transition in a molecule’s CD spectrum, yet our experiments reveal that a change in polarization is observed for the same molecule under different pH* conditions. To ensure that a change in the handedness of the molecule is not responsible for the different CISS responses, CD spectra of l- and d-cysteine were measured under different pH* conditions; see Figure S5. While the CD intensity decreases and the peak modestly red-shifts as pH* increases, the sign of the CD signal does not change. Thus, we conclude that the change in polarization must arise from changes in the surface–cysteine interaction at different pH* rather than any interconversion or racemization of the cysteine enantiomers.

The geometry and packing density of chiral organic ligands on nanoparticle surfaces have been shown in previous studies to affect the resulting chirality of the nanomaterial^33−35^ and therefore we posit that a similar mechanism could give rise to the changes in polarization with pH* here. From the form of the Rosenfeld equation, which relates the rotatory strength to a product of the electronic and magnetic transition dipole moments,^36^ it is clear that the chiroptical response of the cysteine adsorbate and its electronic imprint on the metal substrate can change with adsorbate configuration. Given the low signal-to-noise, experimental CD spectra could not be obtained. While a theoretical determination of the pH-dependent chiroptical response of an adsorbate on a metal surface is possible in principle, it requires a significant development of DFT (or even better, TD-DFT) methods to be accurate enough and is beyond the scope of the current work.^37^ Instead, differences in the geometry of the cysteine adsorbates on the surface were modeled using DFT and used as a starting point to build a model of our data.

**Figure 4:**
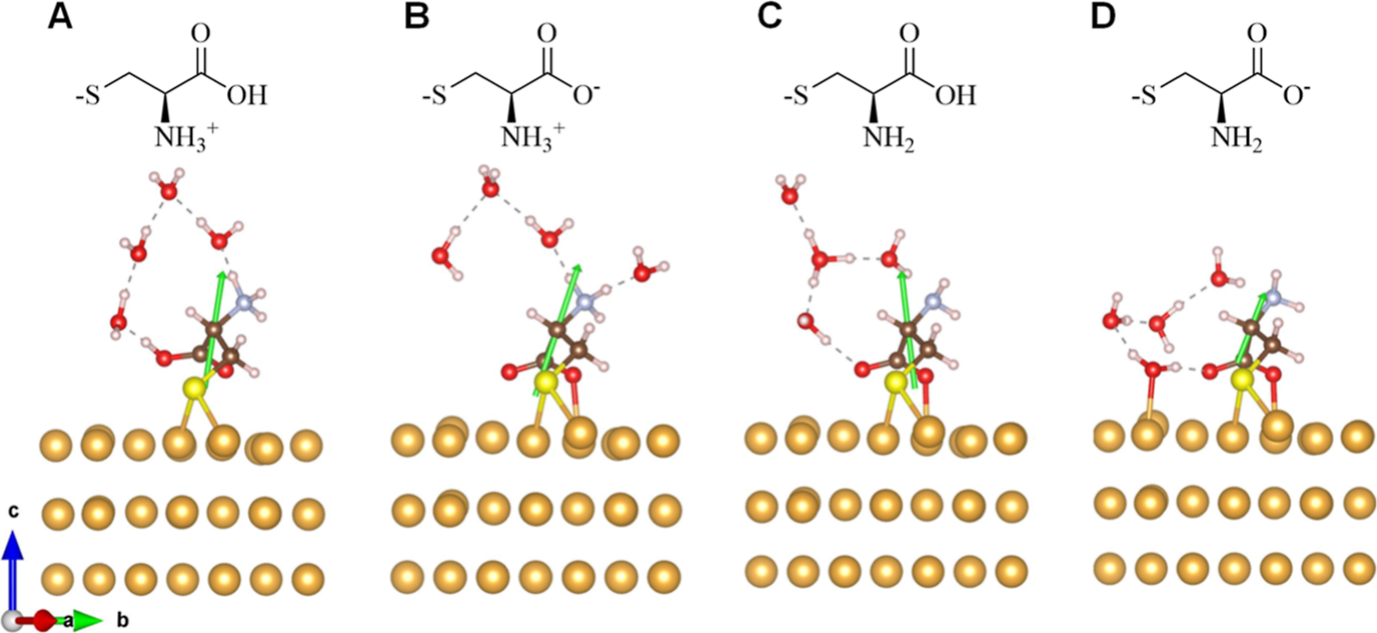
Adsorbate geometries and corresponding dipole moments (green arrow) from DFT calculations determining the most energetically favorable adsorption geometries of an l-cysteine molecule on the surface of Au(111) in H_2_O. The red, livid, brown, and white spheres indicate oxygen atoms, nitrogen atoms, carbon atoms, and hydrogen atoms, respectively. Panel A shows the zwitterionic form of cysteine with a deprotonated thiol group. Panel B shows a thiolate-bound form of zwitterionic cysteine. Panel C shows a tautomer of the species in panel B. Panel D shows a thiolate-bound anionic form of cysteine.

While the gold used in this study is considered polycrystalline, it is important to note that the predominantly exposed facet is Au(111); see Figure S9 and the corresponding discussion in Supporting Information for more details. Now, as discussed above, correlating the changes with pH in Figure 3 to the protonation state of the SH group is likely not very direct because the SH can always deprotonate at a metal surface. Nevertheless, from the data, it would appear that multiple charge states are visited during the course of the experiment. Furthermore, it seems unlikely that, at the pH values and voltage studied here, the cysteine moiety ever has more than a (−2) charge. Therefore, in our simulations below, we will allow for the possibility that the COO group (which is normally deprotonated) can in fact be neutral and protonated at a metal surface, where solvation is more difficult to achieve because of sterics, and the Au–S bonding generates protons. Figure 4 shows the optimized geometry of the four possible ionization states of cysteine, between pH = 8 and 12, in the presence of H_2_O on the Au(111) surface. The geometries of the cysteine adsorbate (neglecting solvent) of the four ionization states are all very similar. However, the distance from the carboxyl group tothe Au surface in panel A differs substantially from that of the other three states (panels B–D); see Table 1 for the distance of the carboxyl group’s oxygen to the gold surface. The structure in panel A shows a strong interaction between the cysteine and the solvent molecules as well, which gives rise to a ring-like system of four H-bonded water molecules. As the ionization state changes, analogous to increasing the pH of the solution, the water molecules in cysteine’s first solvation shell reorganize, causing the ring system to begin to break down.

**Table 1:**
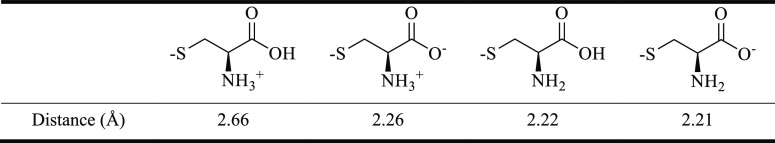
Closest Distance between the Oxygen in the Carboxyl Group to the Au Surface

The change in the geometric structure and hydrogen bonding network correlate with the change in the sign of *P* as shown in Figure 3. Consider the case of l-cysteine; at pH = 8, the adsorption configuration is that of panel A as shown in Figure 4. Because of the formation of the stable 4-H_2_O ring between the carboxyl group and the amine group, the carboxyl group interacts only weakly with the Au substrate, giving rise to the highest polarization value. As the pH increases, however, the population of adsorbate geometries shifts from that of panel 4A to that of panel 4B and the polarization switches sign (goes from positive to negative). In this pH range, the adsorbed cysteine is zwitterionic and its binding geometry changes significantly, which increases and rotates the molecule’s dipole moment. The ring structure is partially destroyed, and the carboxyl group loses its strong interaction with the solvent and instead interacts with the Au substrate. Presumably, these structural and electrostatic changes correlate with changes in the molecule’s chiral imprint on the surface such that the polarization value becomes more negative. As the pH further increases, the adsorbate structures in panels 4C and 4D become more important, the dipole moment decreases (Table 2), the ring structure is distorted, and the carboxyl group interacts more strongly with the Au substrate. Presumably, the distribution of adsorption conformations is such that no significant net polarization is found at high pH and the *P* gradually decreases back to zero. The drop of out-of-plane dipole moment magnitude in panel 4D (also the last column in Table 2) is caused by the absence of a positive charge center and hence a weaker charge separation arises, as compared with the other three states (panel 4A–C). The out-of-plane dipole moment direction, however, does not change (i.e., points along the surface normal) through the whole process, which indicates that the sign change in polarization does not arise from the dipole moment direction but from some other effect of the change in binding geometry. Substituting D_2_O for H_2_O in the calculations does not change the ionization state-dependent structural geometry of the cysteine adsorbate or the H-bonding network (D-bonding network for D_2_O) significantly. The higher mass for the deuterated solvent is known to affect the manifold of vibrational states, i.e., intermolecular vibrational couplings are more important for H_2_O than for D_2_O with a subsequent increase in vibrational energy relaxation.^38,39^ While solvent isotope may affect the strength of coupling between solvent molecules and cysteine, discernible differences in the magnitude of *P* for H_2_O and D_2_O are lacking which suggests that these effects are too weak to elicit a change under the current experimental conditions.

**Table 2:**
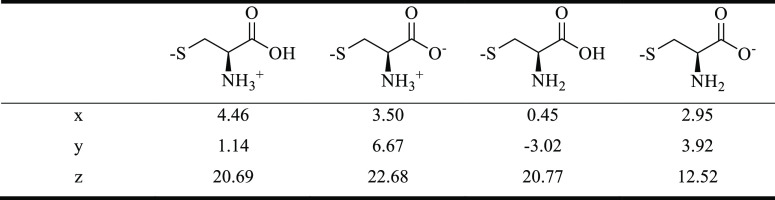
Projection of the Magnitude of Dipole Moment in Cartesian Coordinates *x*, *y*, and *z* Directions for Different Adsorption Configurations of l-Cysteine^a^

a Note that the lattice vectors *a*, *b*, and *c* of the simulation cell constitute a 60° hexagonal cell with *a* in the *x* direction and *c* in the *z* direction.

**Figure 5:**
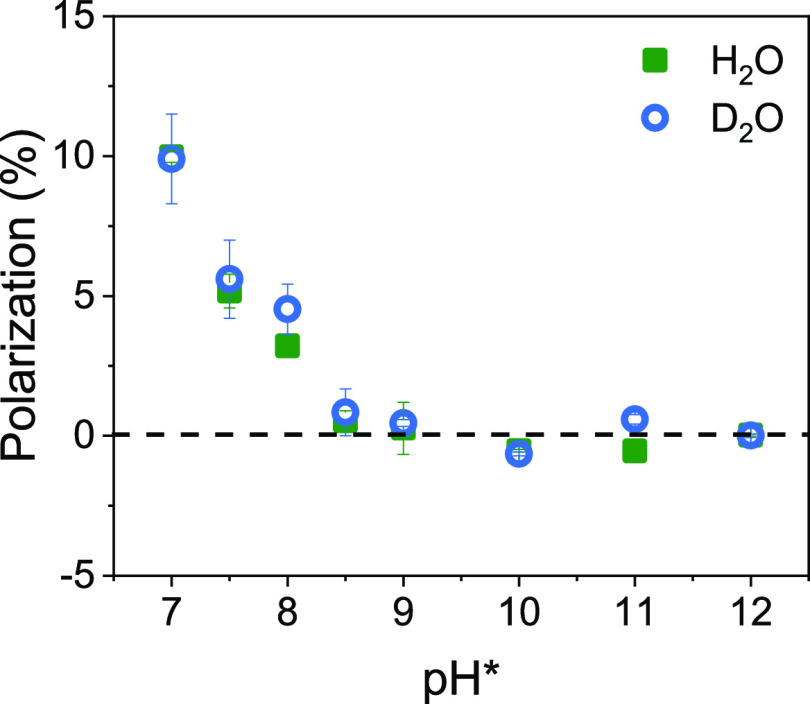
Polarization in the adsorption rate constant of *N*-acetyl-l-cysteine methyl ester in H_2_O (green square) and D_2_O (bluish gray circle) at different pH* values.

**Figure 6:**
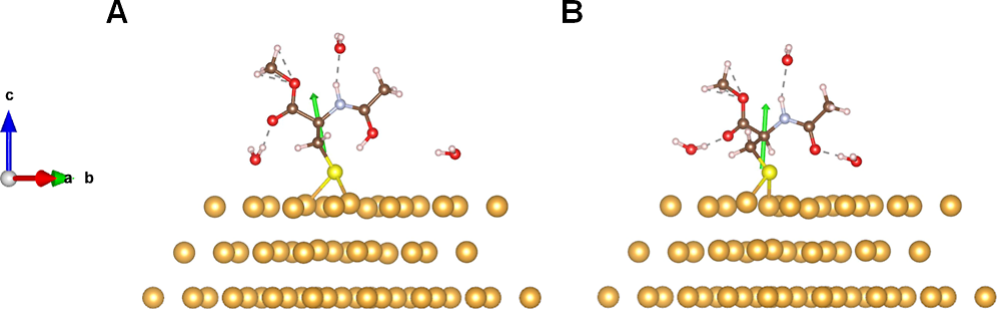
DFT calculations for the most stable adsorption geometry of *N*-acetyl-l-cysteine methyl ester in H_2_O on the Au(111) surface. The red, livid, brown, and white spheres correspond to oxygen, nitrogen, carbon, and hydrogen atoms, respectively. Panel A: before the deprotonation and panel B: after the deprotonation.

The effects of solvent interactions and ionization state on *P* were further explored experimentally using *N*-acetyl-l-cysteine methyl ester as the adsorbate in H_2_O and D_2_O solutions. Note that the carboxylate and the amino groups are both blocked from protonation and their hydrogen bonding interaction with the solvent is weaker; only the thiol group can be deprotonated and its p*K*_a_ in solution is ∼8.5 (though, as discussed above, the relevant proton can always migrate away at an electrode).^40^Figure 5 shows the *P* for 8 μM solutions of *N*-acetyl-l-cysteine methyl ester in H_2_O (green, squares) and D_2_O (blue, circles) as a function pH*. When pH* = 7, the polarization has a positive value of ∼10% in both H_2_O and D_2_O and as the pH* increases to ∼8.5 the polarization gradually decreases to around 0. In contrast to l- and d-cysteine, the *P* for *N*-acetyl-l-cysteine methyl ester does not change sign as pH* increases past the p*K*_a_ associated with deprotonation of sulfur. In addition, no discernible differences in the *P* for H_2_O and D_2_O solutions are evident, i.e., no solvent isotope effect manifests. Figure 6 shows corresponding DFT calculations for the two different possible ionization states of *N*-acetyl-l-cysteine methyl ester; i.e., before and after deprotonation. The optimized geometries show that, similar to the case of cysteine adsorbed on the Au surface, the proton from the thiol group of *N*-acetyl-l-cysteine methyl ester dissociates and bonds to an intramolecular oxygen before the deprotonation. Nevertheless, no specific local solvation structure changes are observed in the deprotonation process, suggesting a weaker interaction to the solvent; this finding is consistent with experiments in which the magnitude of the polarization for *N*-acetyl-l-cysteine methyl ester is smaller than cysteine and no obvious difference between H_2_O and D_2_O are observed.

## Conclusions

This work uses a combination of electrochemistry experiments and DFT calculations to examine the effects of geometric structure, ionization state, dipole orientation, and molecule–solvent interactions on the enantiospecific adsorption of chiral molecules on magnetized ferromagnetic surfaces. Our studies suggest that geometric changes in the adsorbate, brought on by changes in the solution pH, are responsible for changes in the preferred substrate magnetization that is observed for the adsorption of l-cysteine. While DFT calculations suggest that solvent interactions may be important, solvent isotope studies imply that solvent vibrational contributions to the polarization are small and negligible under the current experimental conditions, with the exception of deviations in adsorbate population arising from changes in p*K*_a_ values for H_2_O and D_2_O. Collectively, these studies illustrate some of the considerations necessary for understanding the spin exchange interactions between “simple” chiral molecules and ferromagnetic surfaces which elicit a chiral-induced spin selectivity response.

## Supporting Information Available

EQCM details, adsorption isotherm information, linearization derivation of the isotherm, isotherm data for l- and d-cysteine in D_2_O, experiments and model for derivation of kinetic information, polarization in the adsorption rate constant at different pH values in H_2_O and pD in D_2_O, UV–vis absorbance and CD spectra for l- and d-cysteine in H_2_O and D_2_O, UV–vis and CD spectra for *N*-acetyl-l-cysteine methyl ester in H_2_O and D_2_O, and possible adsorption configurations and dipole moments of l-cysteine in H_2_O on Au in other directions (PDF)
