# Supplementary material for: Spin-Based Chiral Separations and the Importance of Molecule–Solvent Interactions
Source: J Phys Chem C Nanomater Interfaces. 2023 Jul 13;127(29):14155–62. doi: 10.1021/acs.jpcc.3c01159 (PMC10389781; doi:10.1021/acs.jpcc.3c01159)
Supplement: Supplementary file 1 — jp3c01159_si_001.pdf [file jp3c01159_si_001.pdf]

Supporting information

Spin-based Chiral Separations and the Importance of Molecule-solvent Interactions

Yiyang Lu,[1] Tian Qiu,[2] Brian P. Bloom,[1] Joseph E. Subotnik,[2] David H. Waldeck[1*]

[1] Chemistry Department, University of Pittsburgh, Pittsburgh, Pennsylvania 15260, United States.

[2] Departments of Chemistry, University of Pennsylvania, Philadelphia, Pennsylvania 19104, United States

*email: dave@pitt.edu

Contents

Adsorption Isotherm......3

Linearization derivation of isotherm......3

Kinetic model derivation......4

Polarization in adsorption rate constant......5

Abs and CD spectra of cysteine and N-acetyl cysteine methyl ester......5

Underpotential deposition (UPD) of lead......7

The possible adsorption configurations for cysteine and N-acetyl cysteine methyl ester......7

The choice of cysteine orientation......8

The choice of number of explicit water molecules......9

Geometries and energies of sampled structures......10

References......12

Figure S1. Cyclic voltammogram (left) and corresponding frequency change (right) for the electrode in contact with a 20 μg/mL D-cysteine solution in pH 8 phosphate buffer. 3

Figure S2. The left panel shows an adsorption isotherm of D-cysteine using the change in mass determined from the cyclic voltammetry measurements at different cysteine concentrations. The red line is a Langmuir isotherm fit to the data. Right panel shows a linearized Langmuir isotherm plot of the same data in left panel......4

Figure S3. Histogram of the calculated $\Delta_{ads}G$ of D-cysteine (left) and L-cysteine (right) in D_2O at pH* 8.5 for different EQCM electrodes (1−3) under north (red) and south (blue) applied magnetic fields. Studies without a magnetic field on an Au electrode are colored black. The error bars are associated with the error in the linearized isotherm fit......4

Figure S4. Polarization in adsorption rate constant at different pK values (pH for H_2O and pD for D_2O) of solution. L-cysteine (left panel) in H_2O (green square) and D_2O (blue circle). D-cysteine (right panel) in H_2O (purple square) and D_2O (brown circle)....5
Figure S5. Absorbance spectra of 0.4 mM L-cysteine in H_2O (left) and D_2O (right) at different pK values. 5
Figure S6. CD spectra of 0.4 mM L-cysteine (dashed line) and D-cysteine (solid line) in H_2O (left) and D_2O (right) at different pKa values. CD intensities are rescaled based on the absorbance at 200 nm.....6
Figure S7. Absorbance spectra of 0.2 mM N-acetyl cysteine methyl ester in H_2O (left) and D_2O (right) at different pK values....6
Figure S8. CD spectra of 0.2 mM N-acetyl cysteine methyl ester in H_2O (left) and D_2O (right) at different pK values. CD intensities are rescaled based on the absorbance at 200 nm....6
Figure S9. The voltametric (left) and frequency (right) response of the QCM during the underpotential deposition of Pb. 7
Figure S10. Possible adsorption configurations and the dipole moments (green arrow) of L-cysteine in H_2O on a Au based on DFT calculations. The red, gray, brown and white spheres indicate the oxygen atoms, nitrogen atoms, carbon atoms, and hydrogen atoms. Note that this is a side view (in direction of a) of Figure 4 in the main manuscript....8
Figure S11 Possible adsorption configurations and the dipole moments (green arrow) for L-cysteine in H_2O on Au based on DFT calculations. The red, gray, brown and white spheres indicate the oxygen atoms, nitrogen atoms, carbon atoms, and hydrogen atoms. Note that this is a top view of Figure 4 in the main manuscript....8
Figure S12. Possible adsorption configurations and the dipole moments (green arrow) for N-acetyl-L-cysteine methyl ester in H_2O on Au based on DFT calculations. The red, gray, brown and white spheres indicate the oxygen atoms, nitrogen atoms, carbon atoms, and hydrogen atoms. Note that this is a side view (in direction of a) of Figure 6 in the main manuscript....8
Figure S13. Possible adsorption configurations and the dipole moments (green arrow) for N-acetyl-L-cysteine methyl ester in H_2O on Au based on DFT calculatiosn. The red, gray, brown and white spheres indicate the oxygen atoms, nitrogen atoms, carbon atoms, and hydrogen atoms. Note that this is a top view of Figure 6 in the main manuscript. 8
Figure S14. The possible adsorption orientation for cysteine with PCM. A,B,C,D relaxed to E,F,G,H, respectively. The B, C, and D geometries are pre-optimized without PCM, and therefore the geometries do not change much. 9
Figure S15. Optimized geometries of cysteine with explicit water molecules. A, B, and C: cysteine with 3, 4, and 5 explicit water molecules, respectively. Note that with 4 explicit water molecules, a local ring-shape structure forms. This structure effectively unchanges when the 5th water molecule is added....10
Figure S16. Adsorption energy of adding another explicit water molecule. The energy of the 3rd water molecule represents the average adsorption energy of the first three water molecules....10
Figure S17. Several optimized geometries of cysteine with 4 explicit water molecules. Structure D is the most stable structure. 11
Figure S18. Optimized geometries of deprotonated cysteine (-1 H^+) with 4 explicit water molecules. A, B, C, and D have an -NH_3^+ group while E, F, G, and H have an -NH_2 group. The most stable structure among all the sampled geometries is A. In terms of structures that have the -NH_2 group, the most stable structure is F. Note that the proton is delocalized in the water ring in structure F. 11
Figure S 19. Optimized geometries of deprotonated cysteine (-2 H^+) with 4 explicit water molecules. Structure C is the most stable....12

Adsorption Isotherm

Cyclic voltammetry and QCM measurements were used to build the data for the adsorption isotherms. The data were collected by scanning from -0.4 V to - 1.1 V versus saturated Ag|AgCl at a scan rate = 25 mV/s. Unless specified all measurements were made in a pH 8 phosphate buffer solution. The frequency change from the 30th cycle, corresponding to the oxidative adsorption of cysteine on a gold substrate, was converted to a change in mass (Δm) according to the Sauerbrey equation by a factor of - 1.4 ng / Hz. The large number of cycles was needed to allow the instrument to reach equilibrium and give consistent results. Next, the mass change (Δm) or concentration/Δm was plotted versus concentration and fit to a Langmuir – Freundlich isotherm model.

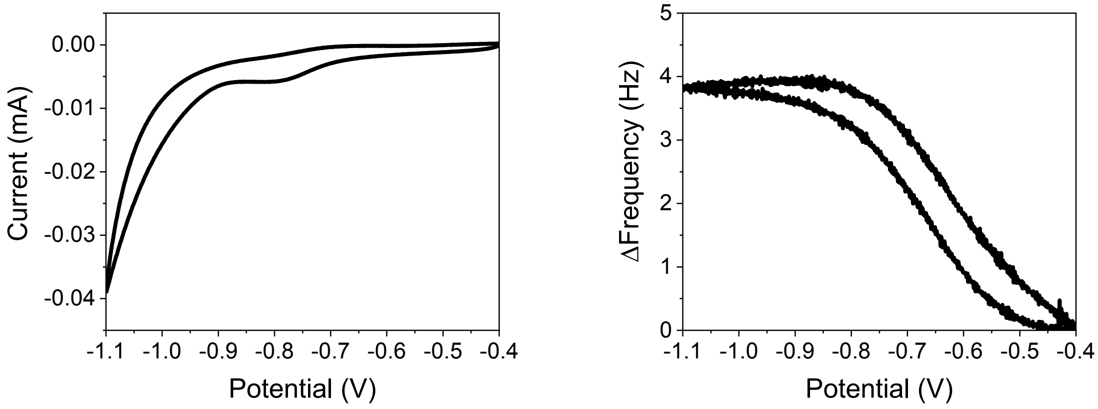

Figure S1. Cyclic voltammogram (left) and corresponding frequency change (right) for the electrode in contact with a 20 µg/mL D-cysteine solution in pH 8 phosphate buffer.

Linearization derivation of isotherm

Assuming that the adsorption is a reversible chemical reaction, the general form of adsorption isotherm can be expressed in a linear form:

$$\frac{c_{cys}}{\Delta m} = c_{cys} \cdot \frac{1}{\Delta m_{max}} + \frac{1}{K_{ads} \cdot \Delta m_{max}}$$

Thus, the maximum adsorption amount and the adsorption equilibrium constant K_{ads} can be calculated by the slope and intercept value of the linear plot. From the K_{ads}, the Gibbs free energy of the adsorption can be calculated by:

$$K_{ads} = \frac{1}{c_{solvent}} \cdot \exp\left(\frac{-\Delta_{ads} G}{RT}\right)$$

where $c_{solvent}$ is the concentration of the solvent, which is usually close to the concentration of pure solvent for low concentration solutions.

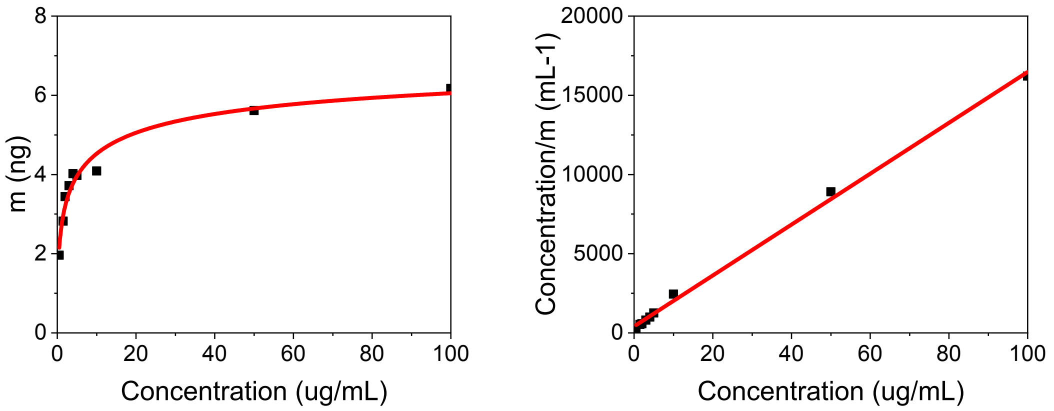

Figure S2. The left panel shows an adsorption isotherm of D-cysteine using the change in mass determined from the cyclic voltammetry measurements at different cysteine concentrations. The red line is a Langmuir isotherm fit to the data. Right panel shows a linearized Langmuir isotherm plot of the same data in left panel.

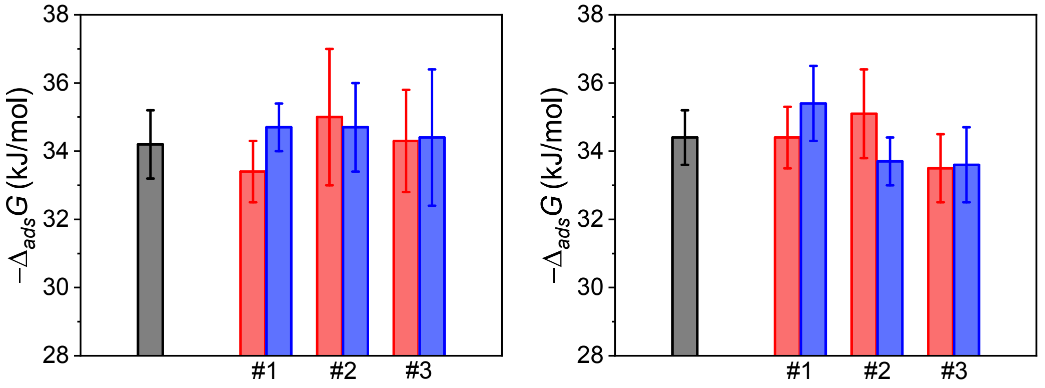

Figure S3. Histogram of the calculated Δ_{ads}G of D-cysteine (left) and L-cysteine (right) in D_2O at pH* 8.5 for different EQCM electrodes (1−3) under north (red) and south (blue) applied magnetic fields. Studies without a magnetic field on an Au electrode are colored black. The error bars are associated with the error in the linearized isotherm fit.

Kinetic model derivation

Assuming a simple Langmuir model, the desorption rate can be written as

$$r_{des} = -\frac{d\theta}{dt} = k_{des} \cdot \theta \quad \text{or} \quad \theta(t) = \theta_{t=0} \cdot \exp\left(-k_{des} \cdot t\right)$$

where θ is the concentration of adsorbed cysteine on the surface (filled surface sites or coverage) and k_{des} is the desorption rate constant. Thus, the rate constant extracted from the exponential fits for the desorption data correspond to k_{des}. For the case of adsorption, we can write the rate as

$$r_{ads} = \frac{d\theta}{dt} = k_{ads} \cdot c_{cys} \cdot (1-\theta) = k'_{ads} \cdot (1-\theta)$$

where c_{cys} is the concentration of cysteine in solution, k_{ads} is the adsorption rate constant, and k'_{ads} is the effective adsorption rate constant.

To quantify the rates for the desorption and adsorption processes, the time responses of the QCM frequency were fit to an exponential growth equation $y = A \cdot e^{t/\tau_1} + y_0$ for desorption and an exponential decay equation $y = A \cdot e^{-t/\tau_2} + y_0$ for adsorption, where A, y_0, and τ_1 and τ_2 were adjusted for a best fit to the data. Then the desorption rate constant was calculated as $k_{des} = \frac{1}{|\tau_1|}$, and the effective adsorption rate constant as $k'_{ads} = \frac{1}{|\tau_2|}$.

Polarization in adsorption rate constant

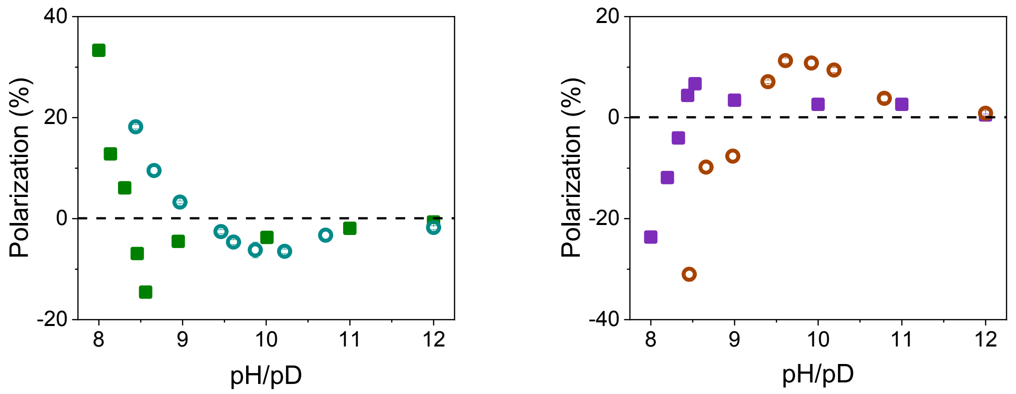

Figure S4. Polarization in adsorption rate constant at different pK values (pH for H_2O and pD for D_2O) of solution. L-cysteine (left panel) in H_2O (green square) and D_2O (blue circle). D-cysteine (right panel) in H_2O (purple square) and D_2O (brown circle).

Abs and CD spectra of cysteine and N-acetyl cysteine methyl ester

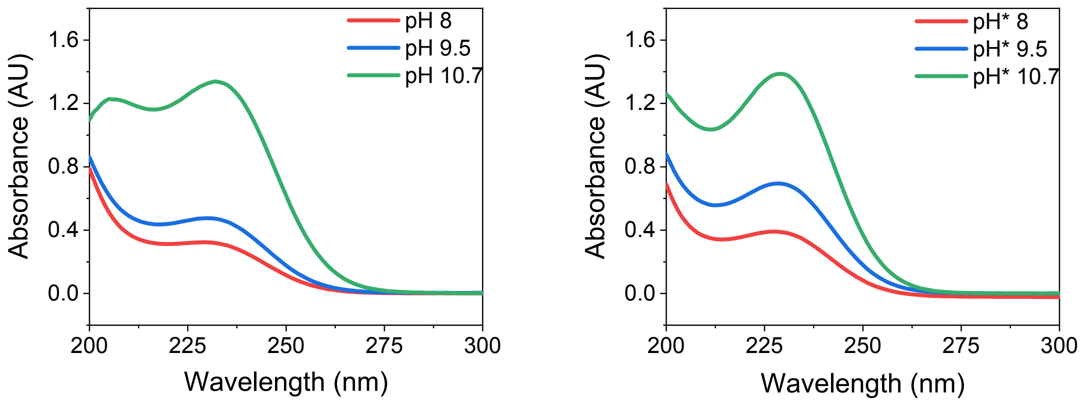

Figure S5. Absorbance spectra of 0.4 mM L-cysteine in H_2O (left) and D_2O (right) at different pK values.

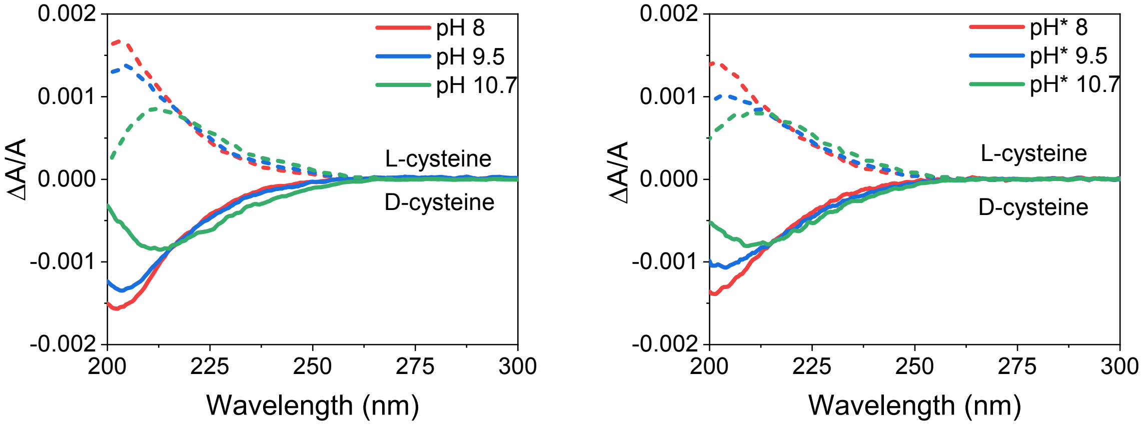

Figure S6. CD spectra of 0.4 mM L-cysteine (dashed line) and D-cysteine (solid line) in H_2O (left) and D_2O (right) at different pKa values. CD intensities are rescaled based on the absorbance at 200 nm.

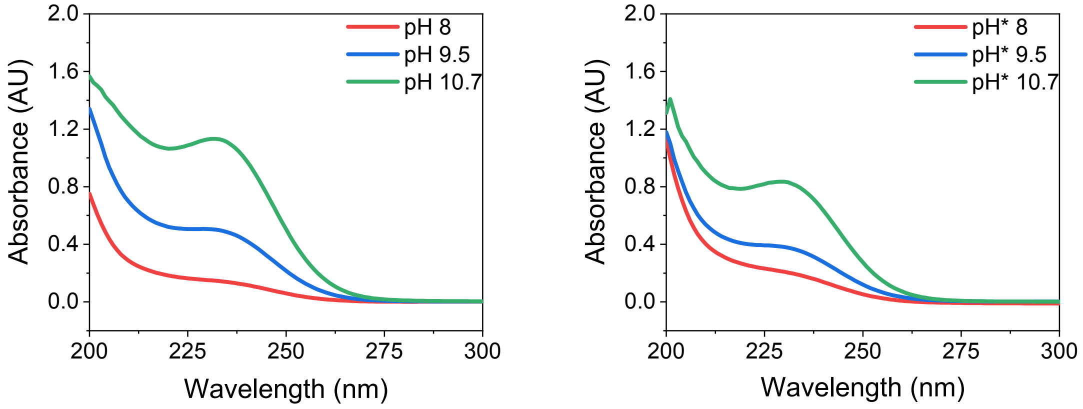

Figure S7. Absorbance spectra of 0.2 mM N-acetyl cysteine methyl ester in H_2O (left) and D_2O (right) at different pK values.

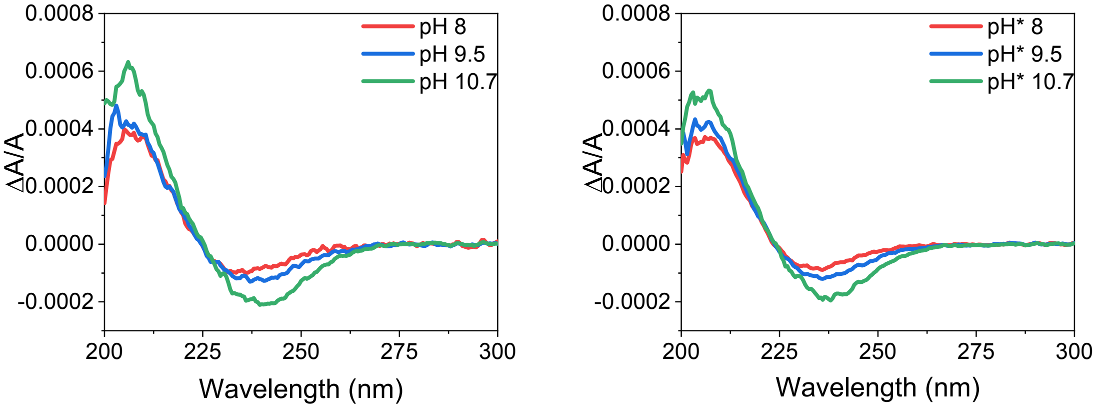

Figure S8. CD spectra of 0.2 mM N-acetyl cysteine methyl ester in H_2O (left) and D_2O (right) at different pK values. CD intensities are rescaled based on the absorbance at 200 nm.

Underpotential deposition (UPD) of lead

Underpotential deposition (UPD) of lead on our substrates were performed using a 5 mM $Pb(ClO_4)_2$, 10 mM of $HClO_4$ and 0.1 M of $KClO_4$ solution. Cyclic voltammograms (left panel) from 0.24 V to -0.45 V versus saturated Ag|AgCl in tandem with the QCM show that the lead adsorbs and gives rise to a cathodic peak at -0.31 V and an anodic peak at around -0.2 V. These redox potentials match well with the reported redox peaks for lead UPD on a Au(111) surface.[1] While the redox peaks using our electrode are broader, associated with other crystalline facets, the most intense, and hence largest contribution, arises from Au (111).

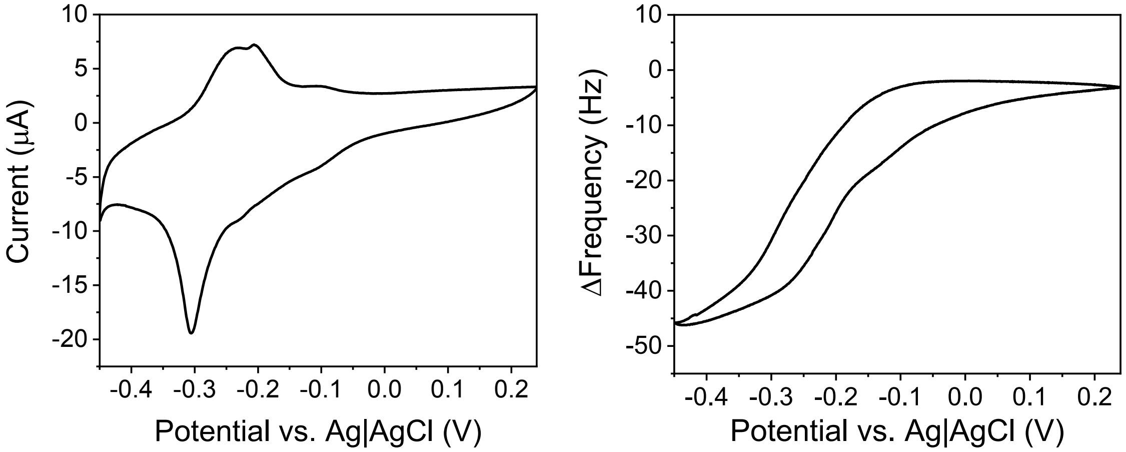

Figure S9. The voltametric (left) and frequency (right) response of the QCM during the underpotential deposition of Pb.

The possible adsorption configurations for cysteine and N-acetyl cysteine methyl ester

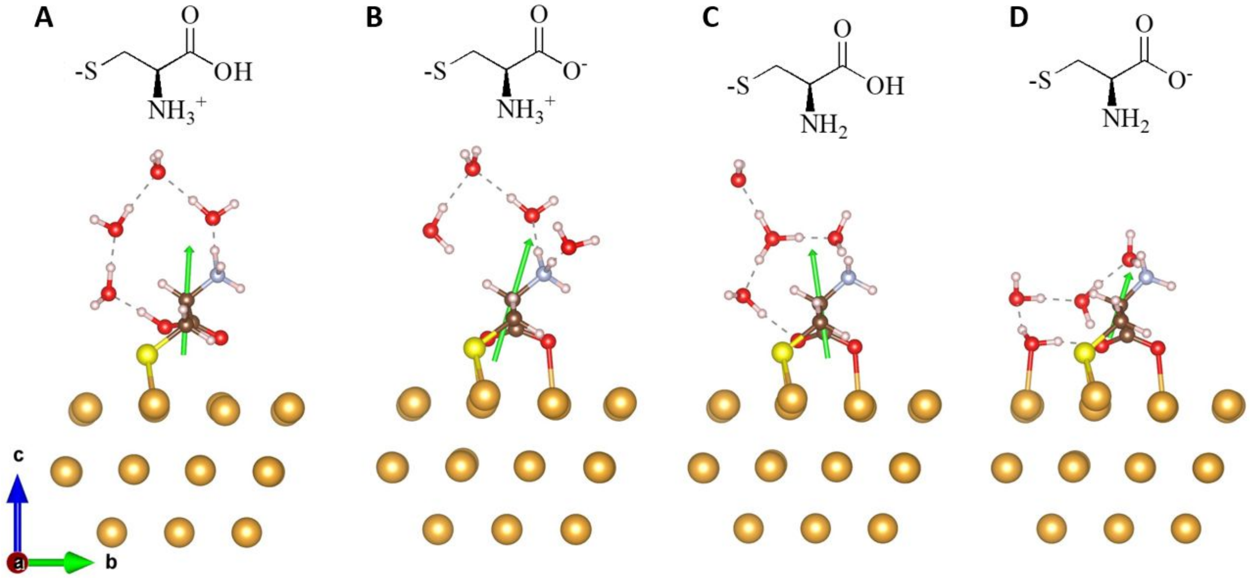

Figure S10. Possible adsorption configurations and the dipole moments (green arrow) of L-cysteine in H_2O on a Au based on DFT calculations. The red, gray, brown and white spheres indicate the oxygen atoms, nitrogen atoms, carbon atoms, and hydrogen atoms. Note that this is a side view (in direction of a) of Figure 4 in the main manuscript.

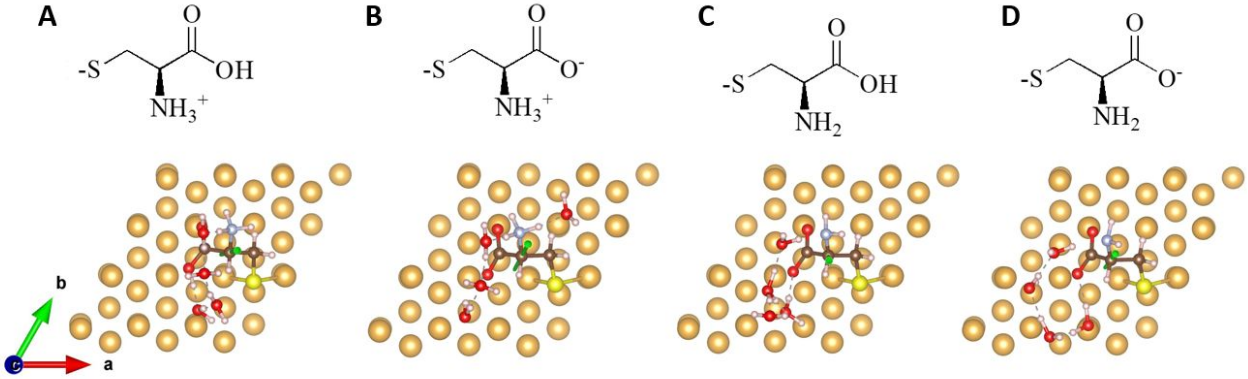

Figure S11 Possible adsorption configurations and the dipole moments (green arrow) for L-cysteine in H_2O on Au based on DFT calculations. The red, gray, brown and white spheres indicate the oxygen atoms, nitrogen atoms, carbon atoms, and hydrogen atoms. Note that this is a top view of Figure 4 in the main manuscript.

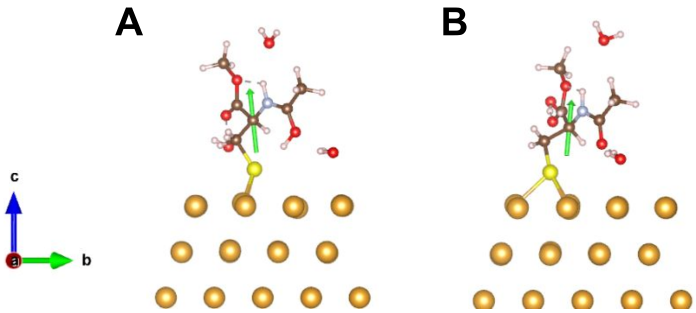

Figure S12. Possible adsorption configurations and the dipole moments (green arrow) for N-acetyl-L-cysteine methyl ester in H_2O on Au based on DFT calculations. The red, gray, brown and white spheres indicate the oxygen atoms, nitrogen atoms, carbon atoms, and hydrogen atoms. Note that this is a side view (in direction of a) of Figure 6 in the main manuscript.

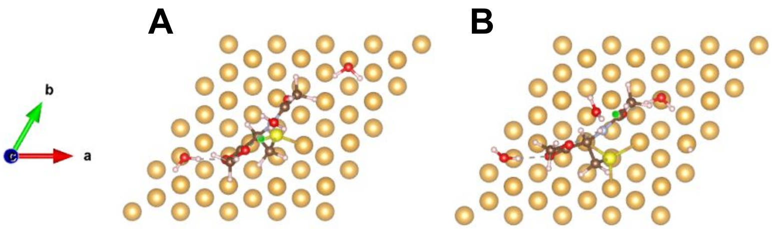

Figure S13. Possible adsorption configurations and the dipole moments (green arrow) for N-acetyl-L-cysteine methyl ester in H_2O on Au based on DFT calculatiosn. The red, gray, brown and white spheres indicate the oxygen atoms, nitrogen atoms, carbon atoms, and hydrogen atoms. Note that this is a top view of Figure 6 in the main manuscript.

The choice of cysteine orientation

We explored a large number of possible geometries for cysteine adsorbed on the Au surface. We began by investigating which functional groups of cysteine are most important in adsorption. We explored geometries

with (A) -COO$^-$, (B) -S$^-$, (C) -SH and -COO$^-$, and (D) -SH, -COOH, and -NH2 interacting with the Au surfaces. Besides the default simulation settings mentioned in the manuscript, a polarizable continuum model (PCM) with the dielectric constant of 80 is also applied by the Environ addon of Quantum Espresso. The geometries before and after the optimizations are shown in Figure S14 and the energies for the optimized geometries are listed below the corresponding geometries.

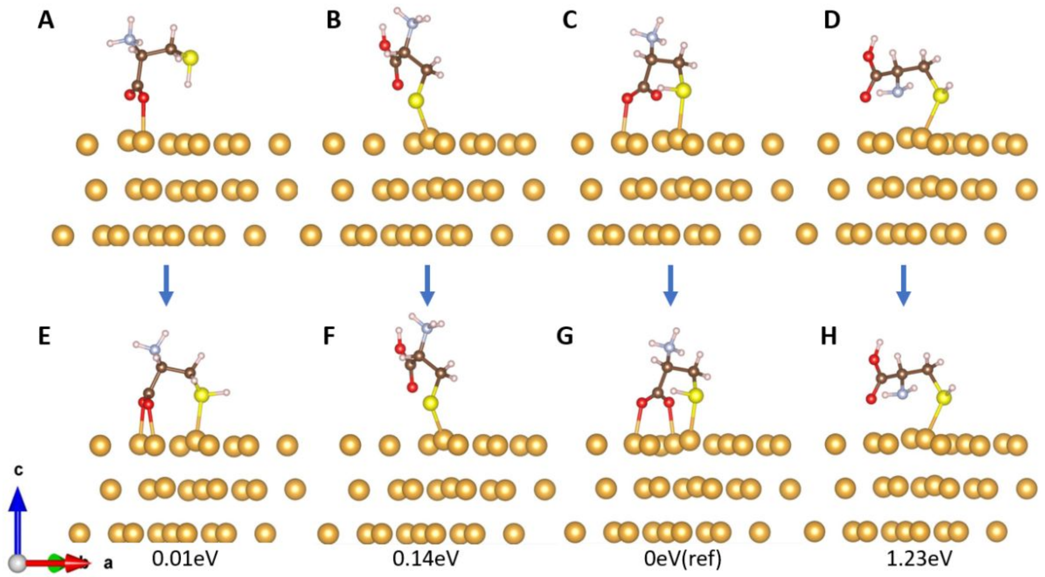

Figure S14. The possible adsorption orientation for cysteine with PCM. A,B,C,D relaxed to E,F,G,H, respectively. The B, C, and D geometries are pre-optimized without PCM, and therefore the geometries do not change much.

The choice of number of explicit water molecules

To investigate whether the local solvation structure is important or not, explicit water molecules have been added to the simulation cell in the presence of PCM. The structures of adding 3,4, and 5 water molecules before and after the optimization are shown in Figure S15. The adsorption energy of added water molecule is calculated as the reaction energy of the following reaction:

$$S_i(PCM) + H_2O(PCM) \rightarrow S_j(PCM)$$

where structure S_j has one extra explicit water molecule than S_i. The adsorption energy of adding the 3rd, 4th, and 5th water molecule is plotted in Figure S16 (the data point for the 3rd water molecule is in fact the averaged adsorption energy of all the first three water molecules). Note that adding the 4th water molecule causes a significant energy drop compared to adding 3 and the 5th water molecule. Also, from Figure S15 B and C, we find that a local water-ring structure forms when the 4th water molecule is added, and the structure is effectively unchanged when the 5th water molecule is added. Consequently, we choose to add 4 explicit water molecules.

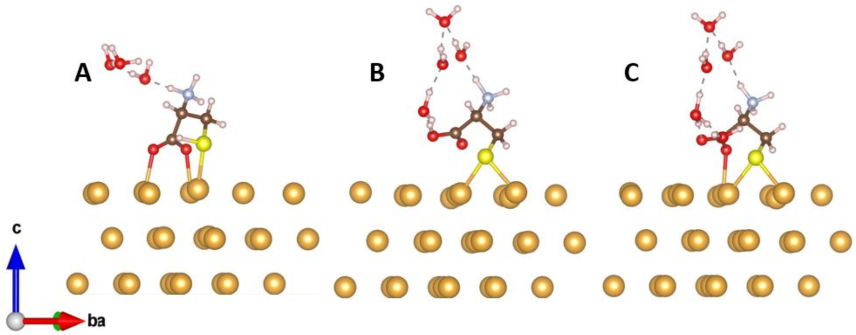

Figure S15. Optimized geometries of cysteine with explicit water molecules. A, B, and C: cysteine with 3, 4, and 5 explicit water molecules, respectively. Note that with 4 explicit water molecules, a local ring-shape structure forms. This structure effectively unchanges when the 5th water molecule is added.

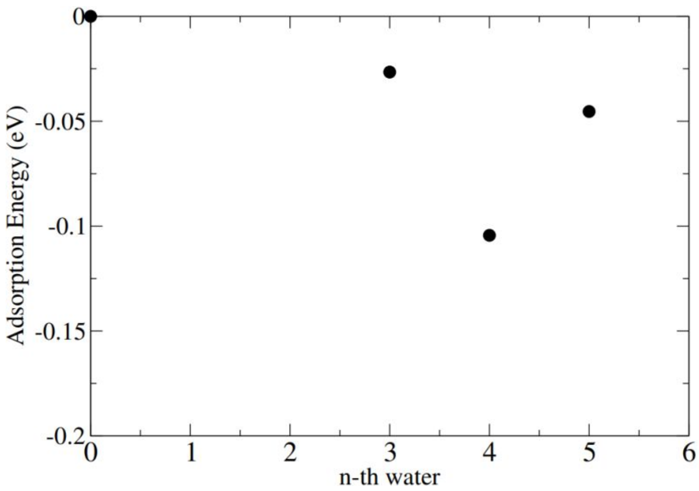

Figure S16. Adsorption energy of adding another explicit water molecule. The energy of the 3rd water molecule represents the average adsorption energy of the first three water molecules.

Geometries and energies of sampled structures

The sampled geometries of cysteine with 4 explicit water molecules after the optimization are shown in Figure S17, and the relative energy of each optimized geometry is listed below the corresponding structure.

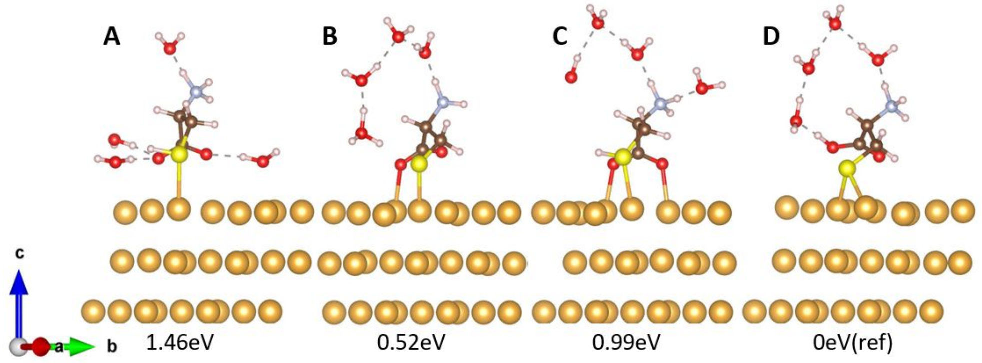

Figure S17. Several optimized geometries of cysteine with 4 explicit water molecules. Structure D is the most stable structure.

The optimized geometries and relative energies of the sampled deprotonated cysteine (-1 H^+) with 4 water molecules are shown in Figure S18. Among all sampled structures in Figure S18, structure A is the most stable one which has the -NH_3^+ group. For structures with the -NH_2 group instead, structure F is the most stable one.

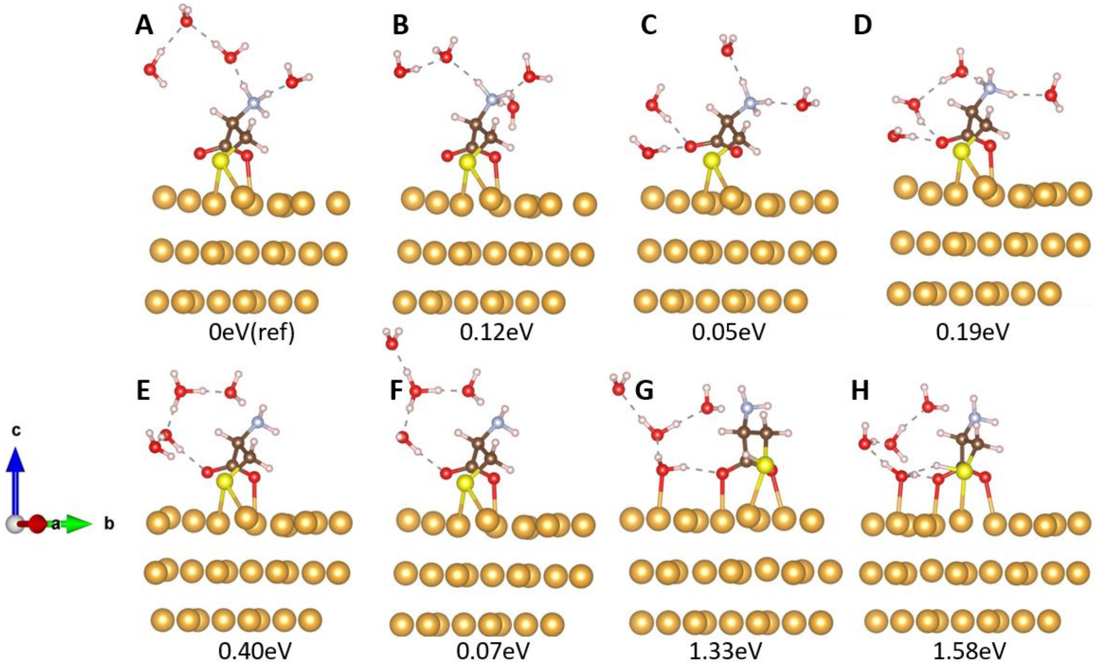

Figure S18. Optimized geometries of deprotonated cysteine (-1 H^+) with 4 explicit water molecules. A, B, C, and D have an -NH_3^+ group while E, F, G, and H have an -NH_2 group. The most stable structure among all the sampled geometries is A. In terms of structures that have the -NH_2 group, the most stable structure is F. Note that the proton is delocalized in the water ring in structure F.

The optimized geometries and relative energies of the sampled deprotonated cysteine (-2 H^+) with 4 water molecules are shown in Figure S19.

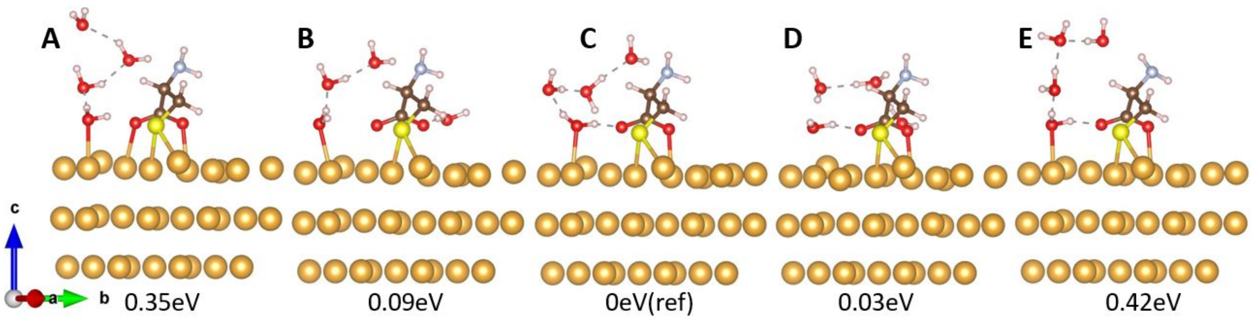

Figure S 19. Optimized geometries of deprotonated cysteine (-2 H^+) with 4 explicit water molecules. Structure C is the most stable.

References

1 Herrero, E., Buller, L. J. & Abruña, H. D. Underpotential deposition at single crystal surfaces of Au, Pt, Ag and other materials. *Chem. Rev.* **2001**, 101, 1897-1930.
